# Key considerations to reduce or address respondent burden in patient-reported outcome (PRO) data collection

**DOI:** 10.1038/s41467-022-33826-4

**Published:** 2022-10-12

**Authors:** Olalekan Lee Aiyegbusi, Jessica Roydhouse, Samantha Cruz Rivera, Paul Kamudoni, Peter Schache, Roger Wilson, Richard Stephens, Melanie Calvert

**Affiliations:** 1 Centre for Patient Reported Outcomes Research, Institute of Applied Health Research, University of Birmingham, Birmingham, UK; 2 National Institute for Health and Care Research (NIHR) Applied Research Collaboration West Midlands, University of Birmingham, Birmingham, UK; 3 NIHR Birmingham Biomedical Research Centre at University Hospitals Birmingham NHS Foundation Trust, Birmingham, UK; 4 NIHR Birmingham-Oxford Blood and Transplant Research Unit (BTRU) in Precision Transplant and Cellular Therapeutics, University of Birmingham, Birmingham, UK; 5 Birmingham Health Partners Centre for Regulatory Science and Innovation, Birmingham, UK; 6 Menzies Institute for Medical Research, University of Tasmania, Hobart, TAS Australia; 7 DEMAND Hub, University of Birmingham, Birmingham, UK; 8 EMD Serono Inc, Healthcare Business of Merck KGaA, Darmstadt, Germany; 9 LAIFE Reply GmbH, Frankfurt, Germany; 10 Patient partner, Centre for Patient Reported Outcomes Research, Institute of Applied Health Research, University of Birmingham, Birmingham, UK; 11 National Institute for Health Research Surgical Reconstruction and Microbiology Research Centre, University of Birmingham, Birmingham, UK; 12 Health Data Research UK, London, UK; 13 UK SPINE, University of Birmingham, Birmingham, UK

**Keywords:** Quality of life, Outcomes research, Clinical trial design

## Abstract

Patient-reported outcomes (PROs) are used in clinical trials to provide evidence of the benefits and risks of interventions from a patient perspective and to inform regulatory decisions and health policy. The collection of PROs in routine practice can facilitate monitoring of patient symptoms; identification of unmet needs; prioritisation and/or tailoring of treatment to the needs of individual patients and inform value-based healthcare initiatives. However, respondent burden needs to be carefully considered and addressed to avoid high rates of missing data and poor reporting of PRO results, which may lead to poor quality data for regulatory decision making and/or clinical care.

## Abstract (web summary)

The collection of patient-reported outcomes (PROs) may capture patients’ assessments of their health status. Here authors highlight PRO-specific issues that should be considered to minimise respondent burden in clinical trials and routine care.

## Introduction

Patient-reported outcomes (PROs) may be defined as “any report of the status of a patient’s health condition that comes directly from the patient, without interpretation of the patient’s response by a clinician or anyone else.”^1^ PROs can provide valuable evidence of the physical and psychosocial impact of disease and treatment on patients’ health-related quality of life (HRQOL) and symptoms^2^. PROs are increasingly used in clinical trials to provide evidence of the benefits and risks of treatments from a patient perspective and to inform regulatory decisions and health policy. The routine collection of PROs, using questionnaires known as patient-reported outcome measures (PROMs), can assist clinicians with the monitoring of patient symptoms; the identification of unmet needs and concerns; the prioritisation and/or the tailoring of treatment to the needs of individual patients and in value-based healthcare initiatives^3^.

Millions of individuals complete PROMs worldwide yearly in a variety of settings. The potential burden of completing PROMs must be considered alongside their benefits. Respondent burden is the degree to which a survey respondent perceives their participation in the project as difficult, time-consuming, or emotionally stressful^4^. The need to justify the benefits of research against the burden and risk is an important ethical consideration^5–7^.

Concerns have been raised about the burden to respondents in completing such measures in clinical trials^1,8,9^ and routine clinical management^10–12^. A recent review of randomised control trials (RCTs) of ovarian cancer reported that compliance rates for PROs were poor overall with levels of preventable missing PRO data ranged from 17% to 41% in the included trials^13^. These poor compliance rates may be due to various reasons including respondent burden. If the issue of respondent burden is not addressed, we risk high rates of missing data and poor reporting of PRO results, meaning poor quality data to inform regulatory decision-making or clinical care^14^. In addition, missing data may be due to various factors, for instance, completion of PROMs may vary depending on ethic/socioeconomic backgrounds^15^. There is a risk that such differences may lead to biased interpretations of trial results or treatment effects in clinical practice.

PRO data from clinical research is frequently underreported or not reported at all, which is unethical^16,17^. A recent study evaluated 160 cancer trials and found that the PRO data of nearly 50,000 participants were never published^17^. If PROs are selected as primary or key secondary outcomes for clinical trials, the findings should be published in the main trial publication. However, if exploratory outcomes the PRO findings may be published in the secondary publication or as supplementary data for the main publication^18^. Furthermore, participant burden may vary across populations, with skilled individuals and those with access to digital technologies being more likely to be able to complete PROMs^15,19^. Failure to address issues of respondent burden whether in relation to electronic or paper PROMs, may further increase health inequalities and risk poorer care in under-served groups^15,19^. For instance, individuals with low literacy levels or cognitive impairment, may find the completion of PROMs burdensome and withdraw from completing the measures^15^. As a result, these individuals may be unable to derive the potential benefits of PRO collection and utilisation in clinical research and routine practice.

Here we identify and highlight PRO-specific issues pertaining to a respondent burden for consideration when planning PRO collection in clinical trials and routine clinical practice.

## Issues pertaining to respondent burden in PRO collection for clinical trials and routine practice

**Fig. 1:**
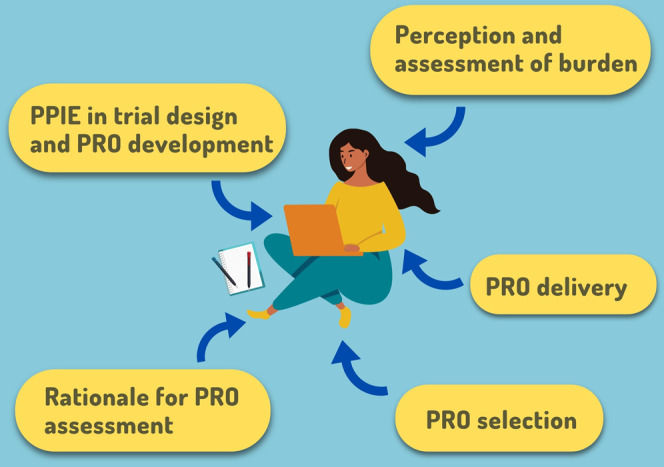
Issues pertaining to respondent burden in PRO collection for clinical trials and routine practice. PPIE patient and public Involvement and Engagement. ©[Chipolla, Studio, Connest byk] via Canva.com.

These include issues relating to the rationale for PRO assessment, measure selection and delivery, patient involvement and engagement (PPIE) in questionnaire development and study design, and the perception and assessment of respondent burden (Fig. 1).

## Rationale for PRO assessment

### Clearly define research objective(s)

The need for clearly defined research objective(s) for PRO data collection, which should inform the selection of the most appropriate PROMs, was highlighted by Shetty et al.^20^. It was also recommended that the formulation of these objectives and subsequent selection of measures should consider patients’ physical and mental capacity and potential respondent burden^20,21^. The collection of PRO data for trials as well as routine care should be evidence-informed to ensure that the data collection justifies the burden and potential risks^22,23^.

### Measuring relevant/useful concepts

The importance of measuring PRO concepts that are relevant and useful to the target patient population was emphasised by Retzer et al.^22^. Static PROMs may include questions that do not apply to sub-groups of patients within a target population^11^. For instance, patients with prostate cancer on hormonal therapy often experience hot flashes and/or breast enlargement which do not occur in those managed conservatively^24^. Vickers et al. reported patients conservatively managed for prostate cancer were asked to complete the Expanded Prostate Cancer Index Composite (EPIC) questionnaire, sometimes left comments such as “I’m not a woman you know” against questions about hot flashes or breast enlargement^24^. There is a potential risk that asking patients to complete irrelevant questions may lead to disengagement and perception of PRO collection as burdensome^25^. In addition, as new therapies are introduced, trialists and clinicians may find legacy measures that have been used for several years in drug development are inadequate for capturing all important symptomatic side effects and other relevant patient experiences^22^. For example, legacy measures do not assess symptomatic side effects from immunotherapy in cancer, which is a limitation for the assessment of the patient experience^26^. Therefore, PROs selected for use in clinical trials and routine practice should be re-evaluated regularly to ensure they remain relevant and appropriate. Possible solutions for addressing the issue of relevance include combining existing measures with new items, the use of item banks or, potentially, the development of new measures^26^.

## Measure selection

### Cognitive requirements

A study that utilised cognitive debriefing to evaluate PROMs in patients with multiple sclerosis found items that required the recall of the frequency of an event or symptom (such as five times per week), which then required categorisation in an ordinal scale, was particularly burdensome for respondents as they required greater cognitive effort^27^. The importance of choosing appropriate recall periods, in terms of the potential burden it can place on respondents, was discussed by a few studies^28,29^. Respondents need to be able to accurately recall changes in their health status without undue strain.

Differences in response burden may also depend on the perceived difficulty of questionnaire completion due to the cognitive requirements of the measure and how unwell the patient may feel^29^. Patients with cognitive impairment are more likely to report a higher response burden when completing PRO measures^29^. In addition, recall periods that are too short may underestimate symptom burden in conditions where symptoms fluctuate diurnally or on a day-to-day basis while recall intervals that are too long may either over- or underestimate patients’ symptoms^28^. Norquist et al. recommended that decisions on the recall period should consider respondent burden alongside other key determinants including the purpose and intended use of the measure, disease characteristics, and the treatment being investigated^28^.

The literacy level required to understand and complete PROMs may also influence respondent burden^30^. It should not be assumed that because patients are engaged in a clinical situation that medical terms can be used and will be understood by patients. It is generally recommended that items be formulated at the sixth-grade reading level or lower; depending on the intended target population and should be justified^30^.

### Number of measures to administer

Often, a single PROM may not capture all the concepts of interest and so may not provide all the vital PRO data required to address all the research questions in a trial or in routine practice^31^. Therefore, it may be necessary to administer more than one PROM. However, trialists and clinical teams need to be careful as the utilisation of multiple PROMs will increase the time required to provide PRO data and may lead to an increase in patient burden^22,32,33^. Therefore, it is important that trialists and clinical teams carefully balance the quantity and quality of data desired, PROM coverage/comprehensiveness and precision against the time requirement and potential respondent burden (including anxiety caused, and fatigue)^23,34,35^. This is important as any increase in patient burden may lead to low compliance, and issues with the integrity and validity of data^22^.

### Questionnaire characteristics

Historically, PROs have relied on long questionnaires which may be more precise but more time-consuming for patients, which are potentially burdensome and may affect compliance^11,23,36,37^. For instance, the original Kidney Disease Quality of Life (KDQOL) questionnaire has 134 items. A shorter 80-item version was developed soon after and a much shorter 36-item version was produced to further reduce potential respondent burden^38^. However, a review and meta-analysis showed that the length of a given PRO questionnaire may not necessarily be associated with participant response burden^29,39^. Furthermore, a feasibility study that used the entire 80 items of the Patient-Reported Outcomes Version of the Common Terminology Criteria for Adverse Events (PRO-CTCAE) Item Library, instead of using a selection of items, reported a high compliance rate^40^. The study suggested that trialists should consider using the entire library, especially for trials of experimental drugs to ensure they adequately capture potential adverse events^40^. Bragstad et al. also highlighted that one challenge with using different short versions of a measure may be the difficulty of comparing the scores of the various versions with each other^41^. Finally, it was noted that while brevity should be a consideration, it should not outweigh the need to assess outcomes that participants consider important^23^.

Despite evidence that the length of the PRO questionnaire may not lead to a burden or affect compliance rates, some authors suggest keeping PRO data collection as brief as possible, without compromising on reliability and validity, especially when patients are very ill or when their condition causes fatigue, lack of energy or tiredness^41–43^. Patient’s health status and functional disability should be considered during the PROM selection process to ensure that length and content are tailored to limit response burden^44,45^. Several studies focused on the development of short forms of existing measures and found that this enhanced the feasibility of collecting PROs on a regular basis and potentially could reduce respondent burden^23,36,41,46–55^. The use of statistical methods such as factor analysis and Rasch can assist with item reduction during the validation of PROMs.

## Measure delivery

### Mode(s) of delivery

Modes of PRO delivery may include paper, smartphone applications, web-based completion, telephone interviews, interactive voice response or audio-computer-assisted interviews^10,22,42,43^. The electronic collection of PROMs (ePROMs) was recommended as an acceptable mode of delivery by several authors^10,34,42,56,57^. A study reported that the use of ePROMs could minimise respondent burden and improve compliance^56^. The majority of participants preferred physician-provided devices while the paper format was the least preferred mode of delivery^56^. Additionally, the majority of those participants who opted for paper did not own an electronic device (smartphone or computer). Another study reported that very few older men enroled for a trial chose ePROMs and opted for paper formats instead^22^. Therefore, irrespective of the mode of delivery consideration should be given to participants’ literacy, ability to utilise technology, cultural and personal needs^43^. It was suggested that patients/study participants should be given a range of modes to choose from^10,57^ as preferences may be determined by various factors including age, computer literacy, access to the internet and electronic devices, and language difficulties all of which may contribute to respondent burden^10,43^. Conversely, the current ISPOR recommendation discourages mixing of paper and electronic PRO collection modes and suggest that mixing of electronic modes may be considered for clinical trials only after equivalence has been established^58^. This issue will be an important consideration/discussion going forward in an era where decentralised trials and BYOD (bring your own devices) are coming to the fore.

The potential impact of developing and collecting ePROMs rather than relying entirely on the traditional paper and pen mode was explored by Retzer et al^22^. Recent technological advances have facilitated the development of ePROMs which were reported as easier and faster for patients to complete thus reducing respondent burden and in turn enhancing compliance with PRO collection^59^.

### ePROM functionalities

A study by Dumais et al. suggested various ePROM features which may enhance compliance by encouraging patient engagement with PRO collection. Increasing engagement could potentially minimise the perception of respondent burden and vice versa^56^. These features include (1) an estimation of ePROM completion time at the outset, (2) the ability to track progress using a progress bar or indicator, (3) graphical presentation of PRO data to personally track their health status, (4) positive messaging to encourage completion when needed, and (5) a thank you message after completion of a daily diary. The study also reported a preference by participants for navigation buttons to be placed at the bottom of the screen and one question presented per screen page as this was considered easier to read^56^. Conversely, a systematic review suggested that presenting one question per screen page may be burdensome for respondents as this format may require more time to click through all the pages to the end of the questionnaire^45^. Work with patient partners and other stakeholders can help in choosing the appropriate PROM functionality for the target population.

### Computerised adaptive testing (CAT)

CAT, which is based on the principles of item response theory, was proposed by several studies as a potential approach for tailoring ePROMs to the individual respondent thereby addressing respondent burden due to the presence of irrelevant questions within a questionnaire^11,37,45,60–82^. Most of the studies utilised the approach in conjunction with the PROMIS item banks.

The use of CAT could enhance the efficiency of PRO collection and significantly reduce the number of questions patients need to answer thus decreasing the time required for completion, without affecting precision or validity^60,63,70,73,76,77,80^. Unlike static forms such as the Hip Disability and Osteoarthritis Outcome Score-Physical Function Short form (HOOS-PS) which requires patients to answer a question about running even if a patient indicates issues with getting in/out of the bath^55^.

### Schedule of assessments

The schedule of assessments will vary substantially by study but should consider participant burden, whilst maximising clinically relevant data to address the research question or clinical use^83^. A qualitative study found no consensus among patients and clinicians on the optimal frequency of administration of PROMs for the routine management of patients with chronic kidney disease^10^. However, interviewees generally believed it would be burdensome if an ePROM was administered more than once a month.

Some studies suggest that brief PROMs for symptom assessment sometimes administered as daily diaries may be burdensome for patients especially those with chronic conditions that require long-term monitoring^32,84^. or who may not experience a significant day-to-day variation of symptoms^28^. Furthermore, the power trialists hope to gain from the additional data obtained from daily administration might be compromised by high levels of missing data potentially due to respondent burden^28,32,84^. Therefore, fewer PRO assessment time points were recommended to minimise respondent burden and optimize resource usage^8,32,45^. Trialists should also consider the nature of the condition and pharmacodynamics of drug interventions when deciding the schedule of PRO assessments^42^. It may be that not all PROMs need to be administered at every time point, depending on the PRO objective. For example, measures of symptom severity could be administered more frequently than measures capturing function or health-related quality of life.

For the routine clinical management of patients, Aiyegbusi et al. suggested that the schedule of assessment would depend on disease trajectory in individual patients, with stable patients requiring less frequent administration^10^. However, deteriorating patients, who may require closer monitoring, might be unwilling to complete PROMs at the level of frequency required due to the burden of illness^10^. It was further suggested that patients should have the option of completing ad-hoc ePROMs if they felt unwell rather than wait for the next scheduled assessment^10^.

Mercieca-Bebber et al recommended that PRO assessments in routine clinical practice should coincide with patients’ clinic visits so that clinicians have the results to review before hospital appointments^45^. The importance of minimising institution/staff burden was also highlighted as an overly burdensome PRO assessment schedule for staff may lead to a reduction in their engagement with the administration and monitoring of compliance rates, thus potentially resulting in high rates of missing data^45^.

### Support for completion

Aiyegbusi et al suggested that PROMs administration for routine clinical practice was best completed at home prior to clinical appointments away from the pressures of busy clinics and potential interference from medical personnel^10^. Although remote/home completion of PROs may be more relevant for routine practice and less so for clinical trials, the growing interest in the use of digitally-enabled decentralised clinical trial (DCT) designs means that remote collection of PROs may become more relevant for clinical trials in future^85^. In clinical research settings, where patients complete paper versions of PROs at home, the provision of postage-paid, self-addressed envelopes may enable the easier return of the questionnaires. However, posting these back to researchers and clinical teams may still be burdensome for some respondents especially those in rural areas^45^. If assessments were to occur in routine clinic/outpatient settings then support for example, provision of childcare (to facilitate completion of PROMs without distractions) and travel assistance (to ensure patients attend their appointments and arrive on time to so they have enough time to complete their PROMs/ePROMs without being rushed) have been suggested^45^.

Patients may not be able to complete PROMs, often through cognitive impairment, or ill-health. In these situations, reports from proxies (typically family or other carers) may be used. Proxy reporting is a longstanding consideration in palliative care research^86^ as well as childhood health research^87^. However, regulatory agencies such as the FDA^1^ and EMA^9^ discourage the use of proxy reports, citing concerns about disparities between proxy and patient reports^1,9^. Nonetheless, in other situations such as PRO data collection for registries^88^ in routine clinical paediatric care^89^, the anticipated need for proxy reporting is recognised. The need for clear guidance on proxy reporting has been noted in palliative care^86^, and there is some guidance for the use of self- or proxy reporting in children and adolescents^90^. Additional work is required to optimise the use of proxy-reported data^91^ as well as adapt existing PROMs or develop new ones for people who have difficulties completing the currently available PROMs (e.g., develop versions with pictures, icons or emoticons).

## Patient involvement in questionnaire development and trial design

During questionnaire development, interviews or focus groups with patients who have lived experiences of the condition of interest can help determine the most appropriate recall intervals for that patient population given the intended use of the PRO measure^28^. In addition, involving patients in cognitive debriefing during the questionnaire, validation could help identify issues with items or formatting which may lead to respondent burden if not addressed^45^.

A patient interviewee who participated in a qualitative study that explored PRO collection and reporting in oncology trials, believed patients need to be involved and share the responsibility for PRO decisions and made to feel like members of the research team^22,92^. Patients can provide feedback on the acceptability and relevance of PRO measures, suitability of assessment time points in capturing desired outcomes, respondent burden, strategies to educate and engage participants, and many other important aspects of study design^45,52^. A study noted that increased consultation with patients at the design phase of clinical trials could lead to the development of a schedule of PRO assessments which may be more relevant for patients and improve completion rates but may not be aligned with clinic visits^21^. However, as previously mentioned, a qualitative study reported that patients were generally in support of aligning the schedule of assessment to clinic visits^10^. Engaging patients and clinicians in study design can help identify a clinically relevant assessment schedule that minimises burden.

## Perception and assessment of respondent burden

### Factors that may influence the perception of burden

Patient comprehension of the purpose of PRO assessment in the context of a trial was mentioned as a potential influence on their perception of burden^42^. Therefore, trialists need to ensure that patients understand the link between their PRO assessments and the questions the trial is trying to answer^42^. Patients also need to understand that substantial missing PRO data would lead to difficulties analysing and interpreting the data which may mean that no useful conclusions can be drawn^22^. These views were corroborated by the qualitative study by Aiyegbusi et al. which reported that patients believed that the provision of explanations of the significance of questions and results would encourage them to complete PROMs on a regular basis for routine clinical care^10^. Poor compliance rates may also occur due to a lack of feedback and utilisation of PROMs results in routine clinical practice. If patients complete PROMs, but the results are not discussed with them or are not seen to be utilised for their care, patients may lose the motivation to complete PROMs over time^10,35,93^.

In clinical trials and routine clinical practice, trialists and clinicians often worry that patients are too ill and so perceive the completion of PROMs as an extra burden that patients may struggle with. The view that a high degree of respondent burden exists (in the absence of any evidence, and often without feedback from patients) might be a reason why some trialists and clinicians are reluctant to facilitate PRO assessments in clinical trials or routine practice^10^. This reluctance could lead to the suboptimal collection of relevant PROs and result in missed opportunities to understand the impact of disease and treatment, which could hamper the design of future trials and routine care. Understanding and implementation of mitigation strategies for respondent burden will ensure that opportunities for collecting relevant and useful data are not missed.

Other factors which may influence the perceived burden of PROs by clinicians include administration requirements, scoring, and how readily the PRO information can be used to inform clinical decision-making as well as the potential impact on clinic workflow is an important consideration, and IT requirements^30^.

### Assessment of respondent burden

A few studies discussed the assessment of respondent burden specific to PROMs^29,94,95^. The number of items could be an indicator for respondent burden^95^. Bryan et al.^94^. mentioned the assessment of respondent burden based on (1) number of items; (2) word count; (3) time for completion (minutes); (4) Flesch–Kincaid grade level (i.e., a measure of readability used to determine how difficult is a text to read).

Atkinson et al. developed the Response Burden Questionnaire^29^, a 6-item measure that assesses how patients perceive: (a) how well the questions related to their actual concerns, (b) how comfortable they were with answering the questions, (c) how well the interview characterised their health and well-being, d) the length of time required to complete the questionnaires, (e) whether questions seemed unimportant or repetitive, and (f) what additional information should have been gathered^29^.

Increasingly, research ethics committees and institutional review boards, in their assessment of PRO research, are considering the burden of PRO collection on study participants^96^. An objective assessment of the potential burden could assist such committees in their decision-making process.

## Key considerations to address respondent burden

Considering and adequately addressing respondent burden relating to PRO collection may ensure that long-term follow-up of patients can be conducted for clinical research and still obtain high-quality data. Avoiding unnecessary burdens is important for integrating PROs into clinical decision-making in routine clinical practice. Efforts should be made to ensure that patients are able to complete PROMs, missing PRO data are minimised and appropriate technology is harnessed to ensure that the data is incorporated effectively and efficiently with existing clinical workflows. It is the responsibility of researchers and clinicians to minimise respondent burden that may arise from completing PROMs.

Key considerations include:

- Having a clearly defined rationale in terms of research objective(s) for PRO data collection should inform the selection of the most appropriate PROMs^20,21^.
- Capturing outcomes that matter to the patient population and involving patients in the selection of measures may mean respondents are willing to complete lengthier measures^10,11,22,42^.
- When selecting PROMs, the characteristics of candidate measures should be considered as these may contribute to potential respondent burden. Various issues as highlighted in the previous sections need to be carefully considered^4,22–24,27–30,32–34,37,41–45^.
- The delivery of PROMs may influence respondent burden. The increasing uptake of technological innovations such as smartphones has facilitated the development and acceptability of ePROMs. A key advantage of utilising ePROMs is that valuable features, impossible with the paper format, can be provided to patients which may help reduce respondent burden and enhance compliance^56^. The collection of ePROMs could also reduce administrative burden and enhance the integration of PROs with existing clinical workflow^59^. However, consideration needs to be given to how to effectively integrate ePROMs into existing workflows and patients should still be given the option of other modes including paper format^10,22,34,42,43,56,57^.
- Computer adaptive testing is a potential approach for tailoring ePROMs to the individual respondent^97^, thereby eliminating the burden due to the presence of irrelevant questions within a questionnaire^37,45,60–77^.
- The schedule of assessment needs to be considered. While the general view is to have fewer assessment timepoints and to align assessments with clinic/study visits^10,32,45^, it is important that patients and clinicians are involved in these decisions. The nature of the condition and effects of drug interventions should also be taken into consideration^28,32,42,84^.
- Early patient involvement and engagement can inform the selection and delivery of PROMs that are acceptable and pose minimal risk of undue burden to trial participants and patients in routine practice^10,21,22,28,45,52^. There is also a need to provide adequate information to patients about the value of PROs in terms of the aims of a research study or routine clinical practice and the implications of incomplete or missing data^10,22,42^. Having this information might provide the motivation patients need to complete PROMs on a regular basis for routine clinical care^10^. Recently, there has been interest in developing ways to measure research participation burden^98–100^. The respondent burden can be assessed regularly and necessary steps are taken to address it^29,94,95^.
